# Translational Health Disparities Research in a Data-Rich World

**DOI:** 10.1089/heq.2019.0042

**Published:** 2019-11-08

**Authors:** Nancy Breen, David Berrigan, James S. Jackson, David W.S. Wong, Frederick B. Wood, Joshua C. Denny, Xinzhi Zhang, Philip E. Bourne

**Affiliations:** ^1^ National Institute on Minority Health and Health Disparities, National Institutes of Health, Bethesda, Maryland.; ^2^ Division of Cancer Control and Population Sciences, National Cancer Institute, Bethesda, Maryland.; ^3^ Institute for Social Research, University of Michigan, Ann Arbor, Michigan.; ^4^ Department of Geography and Geoinformation Science, George Mason University, Fairfax, Virginia.; ^5^ National Library of Medicine, Bethesda, Maryland.; ^6^ Biomedical Informatics and Medicine, Vanderbilt University Medical Center, Nashville, Tennessee.; ^7^ Data Science Institute and Department of Biomedical Engineering, University of Virginia, Charlottesville, Virginia.

**Keywords:** big data, translation, interventions, NIMHD Methods Pillar, AI, algorithmic bias

## Abstract

**Background:** Despite decades of research and interventions, significant health disparities persist. Seventeen years is the estimated time to translate scientific discoveries into public health action. This Narrative Review argues that the translation process could be accelerated if representative data were gathered and used in more innovative and efficient ways.

**Methods:** The National Institute on Minority Health and Health Disparities led a multiyear visioning process to identify research opportunities designed to frame the next decade of research and actions to improve minority health and reduce health disparities. “Big data” was identified as a research opportunity and experts collaborated on a systematic vision of how to use big data both to improve the granularity of information for place-based study and to efficiently translate health disparities research into improved population health. This Narrative Review is the result of that collaboration.

**Results:** Big data could enhance the process of translating scientific findings into reduced health disparities by contributing information at fine spatial and temporal scales suited to interventions. In addition, big data could fill pressing needs for health care system, genomic, and social determinant data to understand mechanisms. Finally, big data could lead to appropriately personalized health care for demographic groups. Rich new resources, including social media, electronic health records, sensor information from digital devices, and crowd-sourced and citizen-collected data, have the potential to complement more traditional data from health surveys, administrative data, and investigator-initiated registries or cohorts. This Narrative Review argues for a renewed focus on translational research cycles to accomplish this continual assessment.

**Conclusion:** The promise of big data extends from etiology research to the evaluation of large-scale interventions and offers the opportunity to accelerate translation of health disparities studies. This data-rich world for health disparities research, however, will require continual assessment for efficacy, ethical rigor, and potential algorithmic or system bias.

## Introduction

Despite decades of research and interventions significant health disparities persist.^1^ Recently, the National Institute on Minority Health and Health Disparities (NIMHD) identified a research framework for understanding causes of health disparities across multiple levels of influence.^2^ However, despite the spurt of health information technology and big data, inadequacies in sample size, collection, and analysis techniques have limited the ability of investigators to understand causes shown in the research framework or to develop and evaluate interventions that can reduce disparities and improve health outcomes.

The National Institutes of Health, led by the NIMHD, and including extramural scientists, initiated a multiyear visioning process to identify gaps and research opportunities.^3^ The process was designed to frame the next decade of research and actions to improve minority health and reduce health disparities. “Big data” was identified as a research opportunity.

A workshop with a range of experts in big data and health disparities was convened on April 22, 2016. A literature review was completed with input from the resulting established writing group. This provided a baseline of current literature in the field. However, most of the ideas were developed by the authors to fill gaps and identify future research opportunities. Rather than a structured literature review, for which there is published guidance,^4^ this narrative review relies on expert opinion designed to provide clarification and insight.^5^

Two research strategies emerged from the workshop, which guide the structure of this narrative review. The first strategy is to foster linkages between traditional and big data sources to magnify data's analytic capacity and more swiftly translate health disparity findings into health disparity reductions. The second is to develop and define best practices for using geographic identifiers in health disparity research to promote place-based research.^3^ Experts collaborated to transform knowledge from a range of disciplines into a more systematic vision of how to use big data to both improve the granularity of information for place-based study and to translate health disparity research efficiently to improve population health.^6^ This Narrative Review is the result of that collaboration.

Interventions on a single determinant cannot eliminate population health disparities.^7^ Health disparities result from a complex causal web involving biology, behaviors, residence, social interactions, and intergenerational inheritance.^8^ For example, we know that racism and economic inequalities interact to cause health disparities, but precisely how these factors interact to cause health disparities in specific places and populations is not clear enough to develop interventions that will reduce resulting disparities.

More knowledge is needed on how race, class, gender, homophobia, and other “isms” drive disparities through mediators such as lack of access to health care or structures that constrain choices and opportunities when using an iterative approach. Systems for the translation of research aimed at reducing health disparities are lacking.^9^ We propose a cyclical translational model to systematically test, evaluate, and adapt proposed interventions. The novelty of this approach resides in combining a cyclical translational model using big data to reduce health disparities.^10–13^

**FIG. 1.**
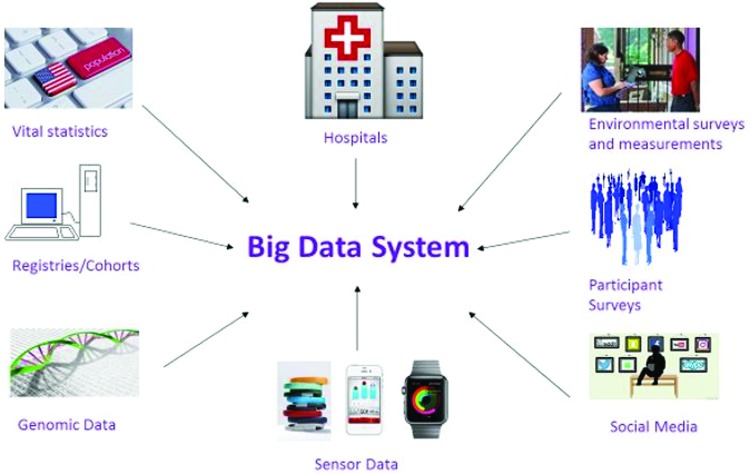
Exemplary data elements for a comprehensive big data system.

A big data system (Fig. 1) can incorporate information from different sources, including vital statistics, registries/cohorts, electronic medical records, household and/or telephone surveys, environmental data genomics, and sensing data from personal devices and social media. The Oxford English Dictionary defines big data as data of a very large size, typically to the extent that its manipulation and management present significant logistical challenges. Because this definition is relative and because our capacity to collect data and to process it is always expanding, it is difficult to define big data with more specificity.^14^ In addition to large volume, big data often are characterized by structural heterogeneity (“variety”) and a torrent of information (“velocity”).^15^

Moreover, what constitutes big data is a moving target. For example, in 2003–2006 the National Health and Nutrition Examination survey (NHANES) collected uniaxial accelerometry data on about 12,000 people at 1-min epochs for 1 week. Already the size of this data set proved a challenge to public health analysts. In 2011–2014, data were sampled at 80 Hz using triaxial accelerometers, resulting in a dataset >10,000 times larger than the 2003–2006 data.

Altogether, if combined into a big data system, heterogenous information could help translate findings from health disparity research into real-world practice while allowing for continuous adaptation and modification to improve outcomes. The two studies that have successfully used big data sources to advance health disparity research combined structured big data from vital statistics with unstructured big data from Google searches.^16,17^ To address the lack of sociodemographic identifiers in the unstructured data, Google searches were organized into geographic units permitting hypothesis testing.

The NHANES accelerometer data described above, with its rich array of socioeconomic identifiers, also could be used to identify disparities and to test disparity hypotheses related to nutrition and health, including reliability between big data and self-reports. Although only few studies have successfully used big data sources to advance health disparity research,^18^ data mining and machine learning (ML), coupled with advances in hardware technology, signal more opportunities for using big data in translational health disparity research.

**FIG. 2.**
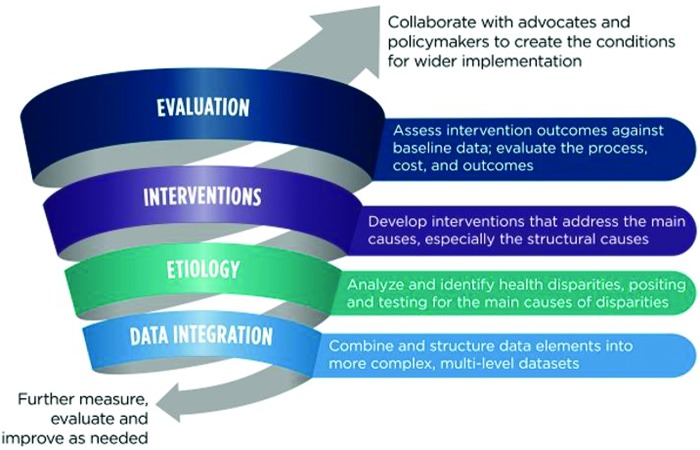
An iterative cyclical approach for reducing health disparities.

An iterative approach (Fig. 2) is proposed with examples of opportunities for translational health disparity research. Most importantly, the approach will require consistent and intense efforts to bring together numerous stakeholders, including researchers from multiple disciplines, administrators and implementers of programs and policies, and representatives of the communities experiencing health disparities to identify data needs and translate findings into real-world settings. Special attention needs to be paid to engaging community representatives and local leaders who will continuously collaborate with researchers to promote change.

Lessons learned from collaborating with community representatives can be used to develop “patient-centered” approaches that can accelerate reductions in health disparities. Patient-centered approaches involve active engagement of patients, and resulting interventions and evidence should both reflect the realities of the diversity of patients and facilitate their adoption of health care decisions in community settings.^19^ Big data collection using collaborative patient-centered approaches in diverse groups also could increase representation of diverse populations. These big data may subsequently be used in training data sets for artificial intelligence (AI) and ML.

So-called “hidden” (perhaps stigmatized) and “hard-to-reach” populations challenge data collection, analysis, and reporting and possible algorithmic bias. Solutions demand interdisciplinary efforts that have already begun. For example, algorithms may be developed for determining when populations are of interest for research and for developing initiatives to address them.^20^ A National Academy workshop subsequently was convened to consider alternative study designs, innovative methodologies for data collection, and innovative statistical techniques for analysis in small population groups.^21^ If big data were deliberately collected using widely available cell phones, it could increase sample size in small populations. However, in the United States, rural Native Americans and Alaska Natives, many of whom live in rural, low-income counties, often do not have cell phone connectivity.^22^

## Evolving Data Science to Address Health Disparities

The 1985 *Report of the Secretary's Task Force on Black and Minority Health* (also known as the “Heckler Report”) dramatically highlighted the greater burden of premature death and illness experienced by racial and ethnic minorities compared to non-Hispanic Whites in the United States.^23^ Subsequently, the federal government established *Healthy People*, the first sustained federal effort to collect data to monitor health disparities.^24^ *Healthy People 2020* added social determinant objectives to focus attention on upstream causes of health inequities.

Despite the critical role of government surveillance data in identifying health disparities, recognition is growing that national surveys are insufficient to document geographically-specific disparities, especially in small populations. In many cases data are needed from a finer spatial scale (e.g., neighborhoods, towns, cities, and counties) using meaningful time frames to adequately document disparities and evaluate programs or policies aimed at addressing the particular disparity of interest.^25^ Such detail is required to better describe individuals' activity spaces and exposure to the built, natural, social, and economic environments that influence behaviors and health outcomes. National estimates from federal surveys do not provide this fine level of detail.

Until 2000, exposure data could be matched with census data at all levels of census geography, from block groups to census regions. Due to the elimination of the long form, which included most of the socioeconomic variables, decennial census data after 2000 can characterize only demographics. The American Community Survey (ACS) was intended to replace the long form. Smaller sample sizes, however, collected over a decade make the ACS estimates less reliable than the previously available decennial census estimates.^26^

ACS limitations make conceptual advances, such as the linking of an “exposome” (i.e., the measure of all of an individual's health-related exposures over the life span) with health outcomes, difficult to realize with federal survey data alone. An exposome would collect environmental exposures and link this information with genomic and population disease data to make it possible to assess how exposures are associated with social determinants. The Public Health Exposome (PHE) Project,^27^ funded by NIMHD and the National Institute for Environmental Health Sciences, offers an example. But, so far, it has not identified causal factors for interventions to reduce health disparities possibly because PHE's county-level measures are at a too coarse geographic unit to reflect the spatial variability of local practices and policies.

Quantitative and qualitative approaches are complementary tools. Using mixed—or multiple—methods may be a more promising approach for understanding how local practices and policies shape health disparities, notably in hidden and hard-to-reach populations, and for identifying plausible causal factors and processes that are relevant to their etiology.^28^

Mixed methods have their strengths and challenges.^29^ Qualitative methods, including interviews, can be used to understand sensitive situations and complex life contexts experienced by vulnerable groups, and this knowledge can be used to develop quantitative instruments that are more sensitive to the meanings and interpretations of respondent reports. Big data research maps large-scale social patterns and qualitative results, which can contribute better understandings at finer grain level of participants' subjective perceptions, feelings, and reasons.^30^ In other words, qualitative research can enhance understanding of results from big data analysis.

## A Big Data Approach to Translate Evidence into Practice

Existing frameworks have emphasized cycles of data- and experience-driven improvement but have often intervened on individuals outside the context of their daily lives. Lacking is a data-driven,^31^ solution-oriented^32^ dynamic system^33^ that incorporates diverse data sources into a framework for translational health disparity research. Data-driven health disparity interventions must be anchored within translational research frameworks and the scope extended to include programs and policies.^34^

To make full use of big data in translational health disparity studies, a blending of data science with health disparity concepts and applications is needed. Social media, crowd-sourced information, electronic health records (EHRs), and mobility and other behavioral information captured by wearable devices could be marshaled to supplement survey and related microdata to better understand health disparities. A wide range of devices collect personal and family data ranging from commercial activity monitors to smart mattresses to Internet-enabled smart speakers that activate appliances and electronic devices. Sensors on these personal devices and the Internet-of-Things technology create large volumes of personal data, often in proprietary formats.

The challenge is how data scientists can work with health scientists to use these large volumes of constantly updated, disparate, and complex personal data to better understand underlying associations and to rapidly translate this knowledge into actions that will reduce health disparities. Ownership of many of these types of devices is often skewed toward higher socioeconomic statuses (especially among early adopters) and more technologically literate populations, which could lead to “algorithmic bias” in analyses or tools using complex data streams.

ML and AI more broadly rely on already-collected data in the analysis process. If measures are limited (e.g., only to race and ethnicity) or databases are biased, then the outcomes similarly will be limited or biased. For example, a review of the genotyping for ancestry information markers for 15 cancer cell lines found that those labeled as White/Caucasian were accurate but that several lines labeled as mixed or African American were badly misclassified.^35^ A review of all genome-wide association studies in 2016 found that 81% of participants in genome-mapping studies were of European decent. Without knowing about variations between populations, the authors concluded that the implications of variations in treatment on different populations cannot be known.^36^

This concern was confirmed in an analysis of germline variation in BRCA genes among over 30,000 Chinese individuals, revealing substantial differences in variants present between Chinese and non-Chinese ethnicities.^37^ Additional novel ancestry-specific associations were confirmed using a new study of nearly 50,000 non-European individuals.^38^ Thus, ML and AI that rely on feeder data likely produce biased results because the input data are biased.

Information about health systems may be gathered in many ways. A widely-used approach is to link EHRs to other types of data collected for the same individuals. EHRs contain a wealth of data on patient characteristics, biometrics, health conditions, disease status, access to health screenings, insurance status, and medications that are relevant to health disparities. However, heterogeneity among providers and EHR software vendors, as well as data fragmentation when patients receive care at different institutions, creates challenges for researchers. Even so, recent research has demonstrated the capability to use EHR data for biological and epidemiological studies.^39^ Studies that have standardized data collections and measures across different health care systems include the Patient-Centered Clinical Research Network (PCORnet),^40^ the Electronic Medical Records and Genomics Network,^41^ and the Observational Health Data Sciences and Informatics (OHDSI) consortium.^42^

To overcome the challenge of data fragmentation, networks like OHDSI and PCORnet are developing common data models to combine and compare EHRs across health service providers.^42,43^ Another example, the Cancer Research Network (CRN),^44^ combines clinical with tumor registry data to evaluate cancer outcomes. An advantage of EHR-linked networks is that they can include a broader range of diverse backgrounds (representing the demographics of those presenting to the hospital) and, thus, are sometimes more inclusive than other traditional research cohorts.

Other types of big data showing promise include passively collected data from Internet search engines and from environmental sensors. Neither type has personal identifiers that allow for linkages to individual characteristics. Nevertheless, analysis of search terms entered by individuals can yield insight into behavior, effect, and attitudes of clusters of people in defined geographic units. Sensors that monitor living environments can provide information on the quality of the local environment.

The large sample size and extensive coverage of the Behavioral Risk Factor Surveillance System (BRFSS)^45^ make it the leading resource for understanding geographically-specific health knowledge, attitudes, and behaviors in the United States. Patterns and clusters found in search engine data might be able to augment the BRFSS and other health surveys to yield more granular detail on knowledge, attitudes, and behaviors than are currently available. In short, big data could improve population coverage and timeliness if combined with survey and administrative data. Supplementing BRFSS and other survey or cohort data in this way may provide useful ways for identifying and elucidating underlying causes of disparities among populations.

Big data may be structured or unstructured. Many large-scale sources, such as population-based data, are highly structured, with defined fields. This is also true of commonly used EHRs, such as billing codes, vital signs, or laboratory results, although encoding and quality can vary significantly within and across EHRs. For instance, a given site can have tens to hundreds of laboratory measurements representing “white blood cell counts,” some representing equivalent values and others differing in site, measurement, units, or other differentiators. Increasingly, these data are being mapped to standard vocabularies.

Other big data, such as Internet search queries, social media data, or narrative notes in the EHR, are unstructured. Analyses require computational techniques that identify patterns, such as ML or natural language processing. Image or waveform data may similarly require ML methods. Advances in computer science, computing, and informatics have made analysis of both structured and unstructured data in large volume possible.

## Opportunities and Challenges for Translational Health Disparity Research in a Data-Rich World

Many different types of big data, such as geospatial, EHR, sensor, and molecular “omics,” are being collected, largely independently (Table 1). Each data type has shown promise for discovery, in elucidating more proximate causes of disease, and suggesting approaches for improving health. Although each data type on its own has contributed to basic health disparity research, the biggest opportunity to improve translational health studies may lie in integrating diverse data types to capture the web of causes of disparities. Significant investments will be required to learn how to integrate multiple types of big data for this purpose. The examples below illustrate opportunities and challenges of six modalities that may be leveraged to enhance translational health disparity research.

**Table 1.** Selected Types of Big Data and Related Challenges to Address Health Disparities

Approach	Target	Critical questions	General references	Sample applications to disparities	Notable challenges
Mobile sensors (e.g., accelerometry)	Physical activity, sleep, sedentary time	Do physical activity and sleep mediate causal pathways and influence health disparities?	Center for Disease Control and Prevention (2018)^45^ Troiano et al. (2008)^62^	Ogilvie et al. (2009)^46^ National Cancer Institute (2019)^47^ Whitt-Glover et al. (2009)^63^ Belcher et al. (2010)^64^	Improving capacity to obtain representative data through crowd sourcing from consumer devices. Engagement of diverse populations.
Geospatial data	Measures of the environment, exposure-related health disparities, behavior and spatial energetics	What exposures from the natural, built, social, and policy environments are associated with health disparities?	Zhang et al. (2017)^18^ Institute of Medicine (2014)^48^ Juarez et al. (2014)^65^ James et al. (2016)^67^	U.S. Department of HHS (2018)^49^ Vayena et al. (2015)^50^ Wilkinson et al. (2016)^51^ Browning et al. (2017)^66^ Oyana et al. (2017)^68^ Baek et al. (2016)^69^	Appropriate spatial and temporal granularity. Uncertain geographic context. Computational challenges. Inadequate conceptual models.
Citizen science initiatives	Enhanced data collection through citizen engagement	Can data collected by citizen scientists be faster, cheaper, and more extensive than data collected through traditional means?	Bartlett et al. (2019)^52^ Den Broeder et al. (2016)^70^	Fuster et al. (2018)^53^ King et al. (2016)^71^	Data quality. Inclusion of diverse contributors.
Social media	Social interactions, education, diffusion	Can convenience samples of social interactions and information seeking behavior help reveal the causes of health disparities?	Tan et al. (2018)^54^ Agniel et al. (2018)^55^ Yoon et al. (2013)^72^ Sinnenberg et al. (2017)^74^	Fleming et al. (2008)^13^ Chae et al. (2015)^73^	Lack of demographic identifiers. Uncertainty about the extent of meaningful knowledge related to addressing health disparities in social media contents.
Electronic health records	Health screenings, diseases, medications, medical exposures	How are variations in access to health services associated with the risk of health disparities	Doria-Rose et al. (2019)^75^ Denny J et al. (2013)^77^ Collins et al. (2014)^79^ Gottesman et al. (2013)^80^	Adams et al. (2017)^76^ Dreyer et al. (2018)^78^	Fragmentation of care across different sites. Variable data access and quality. Permissions to get access. Methods to interpret.
Omics data	Genetics, epigenetics, proteomics, microbiome	What molecular biomarkers are associated with disparities in exposures?	Buolamwini and Gebru (2018)^56^ Manzoni et al. (2018)^81^	Miller (2013)^57^ Kho et al. (2011)^82^	Lack of demographic details in biological data sets

### Linked structured data

Linked records from nearly universal Medicare coverage in the population ages 65 years and older and the National Cancer Institute's (NCI's) Surveillance Epidemiology and End Results (SEER) cancer registry^46^ make it possible for scientists to explore costs and patterns-of-care for older cancer patients. Widely used SEER-Medicare data provide detailed information about Medicare beneficiaries with cancer.^27^ Long-term follow-up in the Medicare population and the legal requirement that cancer diagnoses be reported to the registry yield nearly complete data for studying cancer outcomes in this age group over time.

Moreover, the data are nationally representative and, if pooled over a few years, enable studies of most counties. Estimates of risk factor profiles, screening behaviors, and treatments have been modeled using SEER-Medicare data.^10,11^ Although SEER-Medicare is not specifically designed to study health disparities, more than 11% (213) of all SEER-Medicare publications have studied health disparities.^47^ These publications demonstrate the feasibility of conducting disparity research with integrated data sets.

### Common data elements

The multisite distributed research data developed by the CRN illustrate how data can be more directly aligned with health disparity research.^44^ CRN common data elements are structured in a standardized manner for 11 million enrollees in 14 nonprofit integrated health care delivery organizations. Furthermore, consistent with recent guidelines from the National Academy of Sciences, Engineering, and Medicine for collecting social determinants of health in EHRs,^48^ data for all CRN enrollees are linked to a census-based Neighborhood Socioeconomic Status Index.^44^ Such common data elements permit an increasingly robust understanding of the upstream social determinants of health disparities among the CRN enrollees and, when shared with CRN physicians, help them ascertain causes of health disparities in their patient populations.

### Community science and citizen science data platforms

In community-based participatory research, communities experiencing health disparities collaborate with researchers to identify priority issues and then participate in data gathering, the intervention design, analysis, interpretation, and translation of findings to address disparities. With the evolution of web-based platforms for data sharing, communities can enhance data collection as “citizen scientists” using approaches like those shown in Table 1. Such collaborations among researchers, program managers, and community organizers can strengthen community participation, improve the granularity and detail of data, and help citizen scientists work effectively with researchers to address disparities in their own communities.

### Large cohort studies that include health determinants

New and emerging cohort studies that include possible health determinants could provide powerful new information to explore and address underlying causes of health disparities. The NIH's *All of Us* Research Program^49^ involving the unprecedented linkage of EHR data, genomics, self-report, and sensor-based data elements was formally launched on May 6, 2018. More than 150,000 have fully enrolled to date. Racial/ethnic minorities are more than 50% of the cohort, and more than 75% are characterized as “underrepresented in biomedical research” (e.g., sexual/gender minorities, low income, and rural location).

In addition to molecular and epidemiological discoveries, the cohort should yield tools and infrastructure to advance data collection, linkage, and integrated analyses of big data from multiple domains that will serve to inform future observational and evaluation studies. Because few data sets link biology and social determinants of health, *All of Us* may provide a unique resource to study health disparities. Moreover, *All of Us* could provide follow-up opportunities to study interventions to reduce health disparities in this longitudinal panel-designed study.

### Using data analytics to analyze Internet marketing platforms

Data mining methods may allow data scientists to find patterns in the range of data types described in Table 1, ranging from biological to social structural health determinants. Data mining has been used for genomics, health-related research involving social media, and more recently, health-related image data, but data mining approaches are applicable to any type of large data set and may aid in health disparity research.

For example, studies by Chae et al. used data from Google search logs to assess geographical area racism and to ascertain whether these measures were associated with well-known disparities in black/white mortality and in black birth outcomes.^16,17^ The pervasive and broad use of Google allowed study authors to examine and compare 196 different market areas within the United States, providing much greater granularity than most federal surveys. Data from market areas were linked with federal death and birth records. Compared to Non-Hispanic Whites, one study found that an increase in area racism of one standard deviation was associated with a 6% increase in the rate of all-cause mortality among African Americans. A second study found that each standard deviation increase in area racism was associated with a 6% increase in prevalence of both preterm birth and low birthweight among African Americans. The authors conclude that the Internet-based measure offers a more accurate indicator of racism than do household surveys because people may not want to report racist sentiments in interview settings.

Measures for areas or regions may be useful for exploring controversial social and economic phenomenon such as racism, given possible social desirability response biases in self-report studies. In addition, Internet data could provide measures of behaviors and attitudes of regions or areas. Such big data could examine a single moment or a change over time in identified specific factors that could be targeted to effectively intervene to reduce health disparities.

### Health disparity surveillance

Big data could help improve racial/ethnic minority health and health disparity surveillance by detecting disease outbreaks, assessing health behaviors and attitudes, and identifying adverse reactions to drugs.^50^ As suggested by authors of the Google study of racism and mortality, an individual's digital data may be less filtered than an interview response. Collection and mining online data offer a new data source for health disparity researchers. However, it also raises questions about accuracy and biases and possible limits on the conditions under which the data may be used in health disparity research.

The six approaches discussed above suggest that data collection methods are changing and illustrate opportunities for improving health disparity research analytics using data science techniques. Big data could supplement federal survey and surveillance data to document local disparities and disparities in small populations, reveal the causes of health disparities, and allow evaluation of programs and policies at multiple spatial scales. To combine data types, data need to be accessible and adequately documented with metadata describing underlying elements as proposed through the FAIR (findable, accessible, interoperable, and reusable) principles.^51^

In addition, the field of data science requires consideration of acquisition; engineering; curation and storage; analytics; visualization and dissemination; and ethics, law, policy, and societal impact. Each represents a distinct challenge for the application of data science and big data resources to health disparity research and translation. Mechanisms to promote close collaboration between data and health disparity scientists are needed to maximize the utility of investments in data collection and health disparity research.

### Ethical responsibilities and other challenges

People experiencing health disparities, researchers, program and policy staff, and community leaders addressing disparities present a spectrum of opinions about the value of big data approaches. These range from lack of trust to acceptance to enthusiastic endorsement. Researchers leading studies must be cognizant and respectful of these differences. Moreover, they have the responsibility to ensure that their research does not cause harm to either individuals or communities. A potential source of harm involves intentional or unintentional incorporation of implicit bias into analyses or tools using complex data streams. The examples below emphasize the importance of addressing possible bias for research on health disparities.

Algorithmic bias is well documented in the financial technology sector. A recent review of studies of mortgage loans suggests that algorithmic loan origination may be less biased than face-to-face assessment because it results in fewer rejected applications, but both approaches lead to African American and Latinx customers paying higher interest rates.^52^ A comparison between a logit model and a machine-learning model found that the machine-learning model triangulated almost perfectly the association between race and mortgage default using other borrower characteristics.^53^ This is concerning because race-based housing discrimination is illegal.

These examples suggest that efforts are needed to eliminate bias in training data sets for tools developed through ML and in applications of technology to decision making. Many risk scoring algorithms in the financial, law enforcement, and health sectors are unknown with proprietary or poorly documented software, making it hard to judge if they are discriminatory.

Approaches to audit these algorithms have been developed and efforts to apply these tools in the health sector and encourage transparency are very important,^54^ but health researchers are just beginning to explore algorithmic bias.^55^ Use of technology in the health sector has the potential to reduce discrimination, but improvements are not automatic. For example, face recognition tools are sensitive to the training data sets used in developing recognition algorithms, including those used by Microsoft and IBM. These tools have much higher error rates for women with darker skin than for lighter skinned men because the training sets are overwhelmingly composed of lighter-skinned male subjects.^56^

Biomedical ethics usually is concerned with harm to individuals. Health disparity research requires coupling many different types of data. Doing so increases the risk for individual harm. In addition, communities experiencing health disparities may find that their entire community is stigmatized by research findings that emphasize or overstate negative features. Therefore, health disparity researchers must be mindful of both social and individual ramifications of data and results.

As big data enter minority health and disparity research, a key ethical concern will be the need to ensure that results equally benefit all populations. Ethical dilemmas associated with who should have access to data and mindfulness about the intended or unintended impact of interpretations need to be constantly considered. These ethical issues need to be addressed when data capacity is being built, and not after the fact.

Another challenge is how best to share complex big data and results with study participants. Big data and training data algorithms that are carefully designed to accurately represent the population have the potential to reduce bias in decision making.^57^ Yet care must be taken to proceed in ways that do not risk losing the trust of participants. Given the history of research on racial and gender minorities in the United States, this point is particularly salient for health disparity research because of the large amounts of sensitive personal data in big data resources.

Researchers need to be constantly mindful of ethical issues and address them in ways that promote respect and trust. Pilot studies that prove value before full-scale implementation and efforts to engage community members early in the process are judicious approaches to eliminating algorithmic biases as the use of computer aided approaches intensifies in health care decision making.

## Specific Strategies to Foster Translational Health Disparity Research

Successfully addressing population health disparities involves a partnership between data providers, data analysts, and those who can implement findings and bring them to scale. An example from the prebig data era illustrates the power of partnerships and suggests how partnerships might be built in the future.

In 2002, Delaware was mobilized to address health disparities.^58^ The governor proposed and the legislature fully funded the Delaware Cancer Consortium to reduce high rates of colorectal cancer incidence and mortality, focusing on cancer screening and treatment for the uninsured with an emphasis on addressing disparities between African Americans and Whites. Through 2011, the program navigated more than 10,000 patients through the medical system and performed 5000 colorectal cancer screenings in African American neighborhoods.

Participating clinics carefully monitored screening results and treatment, using state incidence and mortality data. Screening rates for African Americans rose from 48% in 2002 to achieve parity with Whites in 2009 at 74%. Mortality rates from colorectal cancer for African Americans dropped from 31% in 2001 to 18% in 2009, nearly as low as the 17% rate for Whites.

Delaware provides an example where the governor and legislature acted in concert to bring a scientifically proven intervention to scale. This process followed the linear practice that is widely assumed in much health disparity research: the government supported statewide implementation of a proven intervention. However, such support is rare. Usually, investigators document a disparity, develop an intervention, and hope for implementation. More recently, implementation and dissemination researchers have asserted that for translational research, “cyclical, rather than linear, approach is necessary because translating evidence into practice requires attention to real-world settings in which many contextual variables will influence the implementation process.”^9^

Figure 2 illustrates such a cyclical approach, showing the different stages of research associated with identifying and addressing health disparities, from data integration to dissemination and implementation. Data-driven cycles of research, analysis, and evaluation occur at several levels in this model. Data integration is followed by etiological analysis, which may suggest either further intervention or a need to cycle back for data integration and etiological analysis.

For example, analysis of big data may help identify the intervention “target,” perhaps doctors who are discriminating against some patient groups. Behavioral scientists need to decide what is the best intervention to address this issue. However, interventions need to be embedded in an iterative approach with the capacity, if the intervention is not successful, to adjust and try again. Interventions that successfully address the specific causes discovered by etiological studies should be widely implemented. Interventions that are not successful need to be returned to earlier stages for refinement. The refinement could be a better understanding of possible causation or improvements in the effectiveness of the intervention. Each subsequent cycle validates the previous cycle and guides modifications.

The process illustrated in Figure 2 also represents a larger cycle connecting population surveillance and widespread implementation of proven interventions, programs, and policies. A program that is successful in reducing disparities leads to a new cycle of measurement and a new series of data-driven efforts to target remaining disparities. Monitoring reduction in disparities, especially at the local community level, will depend on access to and clever integration of a wide range of data types. Moreover, data fed into models will need to accurately represent population subgroups to avoid unintended consequences.

Data fed into machines reflect the history of our own unequal society—in effect, asking the program to learn our own biases. To maximize gains in developing actionable evidence and effective interventions to reduce health disparities, information on health disparity populations will need to be accurate. Otherwise, some scholars worry that precision medicine may exacerbate bias in favor of well-off white men.^59^

Table 2 offers strategies for adaptations to each step in this cyclical approach. Arguably the most pressing need is to train a workforce in the translational and data-driven aspects of health disparity research. Collaborative efforts among communities, government, academic institutions, and funding agencies are needed. Already, academia is ramping up data science programs to meet societal demand. Training programs that support a data science concentration in health disparities are also needed.^60^

**Table 2.** Strategies for Applying a Cyclical Approach to Reducing Health Disparities

Overall
Train a multidisciplinary research workforce that includes researchers who are health disparity subject matter specialists and researchers who can iteratively integrate big and other data, apply data science, and translate and visualize results.
Establish organizational structures to involve all stakeholders on an ongoing basis.
Promote a data-driven iterative approach to identifying and mitigating health disparities.
Adapt the “learning healthcare systems” approach to focus on health disparity research.
Engage social entrepreneurs and information technology, data science, and other sectors not traditionally engaged with health disparities.
Collaborate in ways that does no harm to individuals or communities and builds mutual understanding, respect, and trust.
Data Integration and Etiology
Develop data science laboratories that can conduct health disparity simulation/complex systems modeling.
Incorporate features and parameters related to health disparities into electronic health record systems.
Identify and make available reference data sets that can be reused according to the FAIR principles.
Ensure data quality and integrity (e.g., align definitions of race and ethnicity) before data aggregation and analysis.
Interventions
Develop outreach mechanisms that fully discuss and illustrate interventions to build community trust.
Pilot interventions before full-scale implementation, considering ethical and cultural issues.
Evaluation
Conduct scientific evaluation (e.g., hypothesis testing) throughout the process.
Review progress with respect to the NIMHD Research Framework^2^ and recommend actions relevant to the framework.
Conduct iterative process evaluation.
Review cost benefit of big data driven translational research cycles against traditional forms of health disparity intervention research and development.

FAIR, findable, accessible, interoperable, and reusable; NIMHD, National Institute on Minority Health and Health Disparities.

## Conclusion

Translation from bench science to real-world practice generally averages 17 years.^61^ To accelerate translational health disparity research, this narrative argues for an iterative approach driven by big data that involves all stakeholders. Today, unprecedented opportunities exist to broaden the field of health disparity enquiry using a continuously growing spectrum of diverse and novel data sources which, with the right workforce and tools, could lead to greater knowledge about causes of health disparities and more effective methods for addressing disparities than previously imagined. However, a big data-driven cyclical approach will be challenging. The workforce and financial resource are currently limited, and, as with many areas of data science, disparity data are complex, incomplete, lack standardization, and present ethical challenges. Moreover, rapidly translating research findings into interventions requires diverse stakeholders, including communities, the public, industry, academia, and all levels of government, to be engaged throughout all phases of the process.

## Abbreviations Used

AI: Artificial Intelligence
AIMs: ancestry information markers
ACS: American Community Survey
BRFSS: Behavioral Risk Factor Surveillance System
CRN: Cancer Research Network
EHRs: electronic health records
FAIR: findable, accessible, interoperable, and reusable
ML: machine learning
NCI: National Cancer Institute
NLM: National Library of Medicine
NHANES: National Health and Nutrition Examination survey
NIMHD: National Institute on Minority Health and Health Disparities
OHDSI: Observational Health Data Sciences and Informatics
PCORnet: Patient-Centered Clinical Research Network
PHE: Public Health Exposome
SEER: Surveillance Epidemiology and End Results

## References

[1] Agency for Research and Healthcare Quality. National Healthcare Quality and Disparities Report 2017. 2018 Available at www.ahrq.gov/research/findings/nhqrdr/nhqdr17/index.html Accessed 930, 2019

[2] Alvidrez J, Castille D, Laude Sharp M, et al. The NIMHD research framework for minority health and health disparities. AJPH 2019;109:216–S20 10.2105/AJPH.2018.304883 PMC6356129 30699025

[3] Jones NL, Breen N, Das R, et al. Cross-cutting themes to advance the science of minority health and health disparities. Am J Public Health. 2019;109(S1):S21–S24 30699031 10.2105/AJPH.2019.304950 PMC6356138

[4] Green BN, Johnson CD, Adams A Writing narrative literature reviews for peer-reviewed journals: secrets of the trade. J Chiropr Med. 2006;5:101–117 19674681 10.1016/S0899-3467(07)60142-6 PMC2647067

[5] Greenhalgh T, Thorne S, Malterud K Time to challenge the spurious hierarchy of systematic over narrative reviews? Eur J Clin Investig. 2018;48:e12931 29578574 10.1111/eci.12931 PMC6001568

[6] Breen N, Jackson JS, Wood F, et al. Translational health disparities research in a data-rich world. Am J Public Health. 2019;109(S1):S41–S42 30699034 10.2105/AJPH.2019.304951 PMC6356140

[7] Woolf SH, Braveman P Where health disparities begin: the role of social and economic determinants—and why current policies may make matters worse. Health Affairs (Project Hope) 2011;30:1852–1859 21976326 10.1377/hlthaff.2011.0685

[8] Krieger N Inheritance and Health: what Really Matters? Am J Public Health. 2018;108:606–607 29617613 10.2105/AJPH.2018.304353 PMC5888059

[9] Gonzales R, Handley MA, Ackerman S, et al. A framework for training health professionals in implementation and dissemination science. Acad Med. 2012;87:271–278 22373617 10.1097/ACM.0b013e3182449d33 PMC3307591

[10] Dreyer MS, Nattinger AB, McGinley EL, et al. Socioeconomic status and breast cancer treatment. Breast Cancer Res Treat. 2018;167:1–8 28884392 10.1007/s10549-017-4490-3 PMC5790605

[11] Burnett-Hartman AN, Adams SV, Bansal A, et al. Indian Health Service Care System and Cancer Stage in American Indians and Alaska Natives. J Health Care Poor Underserved. 2018;29:245–252 29503298 10.1353/hpu.2018.0017 PMC13183385

[12] IBM. Watson Health. 2018 IBM Watson Health. Available at www.ibm.com/watson/health Accessed 930, 2019

[13] Fleming ES, Perkins J, Easa D, et al. The role of translational research in addressing health disparities: a conceptual framework. Ethn Dis. 2008;18(2 Suppl 2):S2-155–160 PMC2705204 18646340

[14] Press G. 12 Big Data Definitions: What's Yours. Forbes, 2014

[15] Gandomi A, Haider M Beyond the hype: big data concepts, methods, and analytics. Int J Inf Manage. 2015;35:137–144

[16] Chae DH, Clouston S, Hatzenbuehler ML, et al. Association between an Internet-based measure of area racism and Black mortality. PLoS One. 2015;10:e0122963 25909964 10.1371/journal.pone.0122963 PMC4409363

[17] Chae DH, Clouston S, Martz CD, et al. Area racism and birth outcomes among Blacks in the United States. Soc Sci Med (1982) 2018;199:49–55 10.1016/j.socscimed.2017.04.019 PMC5640467 28454665

[18] Zhang X, Perez-Stable EJ, Bourne PE, et al. Big data science: opportunities and challenges to address minority health and health disparities in the 21st century. Ethn Dis. 2017;27:95–106 28439179 10.18865/ed.27.2.95 PMC5398183

[19] Sofolahan-Oladeinde Y, Mullins CD, Baquet CR Using community-based participatory research in patient-centered outcomes research to address health disparities in under-represented communities. J Comp Eff Res. 2015;4:515–523 26436953 10.2217/cer.15.31

[20] Srinivasan S, Moser RP, Willis G, et al. Small is essential: importance of subpopulation research in cancer control. Am J Public Health. 2015;105 Suppl 3:S371–S373 25905825 10.2105/AJPH.2014.302267 PMC4455491

[21] Kirkendall N, White J Improving Health Research on Small Populations: Proceedings of a Workshop. Washingto, DC: National Academies Press, 2018 Available at https://www.census.gov/library/stories/2018/12/rural-and-lower-income-counties-lag-nation-internet-subscription.html Accessed 1025, 2019 29738210

[22] Martin MJR Rural and Lower-Income Counties Lag Nation in Internet Subscription. 2018

[23] Heckler MM In: Report of the Secretary's Task Force Report on Black and Minority Health Volume I: Executive Summary. Edited by U.S. Department of Health and Human Services. Washington, D.C.: Government Printing Office, 1985, p.37

[24] Reed M, Huang J, Brand R, et al. Implementation of an outpatient electronic health record and emergency department visits, hospitalizations, and office visits among patients with diabetes. JAMA. 2013;310:1060–1065 24026601 10.1001/jama.2013.276733 PMC4503235

[25] Park YM, Kwan MP Multi-contextual segregation and environmental justice research: toward fine-scale spatiotemporal approaches. Int J Environ Res Public Health. 2017;14 pii: 10.3390/ijerph14101205 PMC5664706 28994744

[26] National Research Council. Using the American Community Survey: Benefits and Challenges. Washington, D.C.: The National Academies Press, 2007

[27] Juarez PD, Matthews-Juarez P, Hood DB, et al. The public health exposome: a population-based, exposure science approach to health disparities research. Int J Environ Res Public Health. 2014;11:12866–12895 25514145 10.3390/ijerph111212866 PMC4276651

[28] Jeffries N, Zaslavsky AM, Diez Roux AV, et al. Methodological approaches to understanding causes of health disparities. Am J Public Health. 2019;109(S1):S28–S33 30699015 10.2105/AJPH.2018.304843 PMC6356121

[29] Stewart M, Makwarimba E, Barnfather A, et al. Researching reducing health disparities: mixed-methods approaches. Soc Sci Med (1982) 2008;66:1406–1417 10.1016/j.socscimed.2007.11.021 18248867

[30] Mills KA What are the threats and potentials of big data for qualitative research? Qual Res. 2018;18:591–603

[31] Glasgow RE, Vogt TM, Boles SM Evaluating the public health impact of health promotion interventions: the RE-AIM framework. Am J Public Health. 1999;89:1322–1327 10474547 10.2105/ajph.89.9.1322 PMC1508772

[32] Watts DJ Should social science be more solution-oriented? Nat Hum Behav. 2017;1:0015

[33] Spruijt-Metz D, Hekler E, Saranummi N, et al. Building new computational models to support health behavior change and maintenance: new opportunities in behavioral research. Transl Behav Med. 2015;5:335–346 26327939 10.1007/s13142-015-0324-1 PMC4537459

[34] King DK, Glasgow RE, Leeman-Castillo B Reaiming RE-AIM: using the model to plan, implement, and evaluate the effects of environmental change approaches to enhancing population health. Am J Public Health. 2010;100:2076–2084 20864705 10.2105/AJPH.2009.190959 PMC2951937

[35] Hooker SE Jr., Woods-Burnham L, Bathina M, et al. Genetic ancestry analysis reveals misclassification of commonly used cancer cell Lines. Cancer Epidemiol Biomarkers Prev. 2019;28:1003–1009 30787054 10.1158/1055-9965.EPI-18-1132 PMC6548687

[36] Popejoy AB, Fullerton SM Genomics is failing on diversity. Nature. 2016;538:161–164 27734877 10.1038/538161a PMC5089703

[37] Bhaskaran SP, Chandratre K, Gupta H, et al. Germline variation in BRCA1/2 is highly ethnic-specific: evidence from over 30,000 Chinese hereditary breast and ovarian cancer patients. Int J Cancer. 2019;145:962–973 30702160 10.1002/ijc.32176 PMC6617753

[38] Wojcik GL, Graff M, Nishimura KK, et al. Genetic analyses of diverse populations improves discovery for complex traits. Nature 2019;570:514–518 31217584 10.1038/s41586-019-1310-4 PMC6785182

[39] Denny JC, Bastarache L, Ritchie MD, et al. Systematic comparison of phenome-wide association study of electronic medical record data and genome-wide association study data. Nat Biotechnol. 2013;31:1102 24270849 10.1038/nbt.2749 PMC3969265

[40] Collins FS, Hudson KL, Briggs JP, et al. PCORnet: turning a dream into reality. J Am Med Inform Assoc 2014;21:576–577 24821744 10.1136/amiajnl-2014-002864 PMC4078299

[41] Gottesman O, Kuivaniemi H, Tromp G, et al. The Electronic Medical Records and Genomics (eMERGE) Network: past, present, and future. Genet Med. 2013;15:761–771 23743551 10.1038/gim.2013.72 PMC3795928

[42] Hripcsak G, Ryan PB, Duke JD, et al. Characterizing treatment pathways at scale using the OHDSI network. Proc Natl Acad Sci U S A. 2016;113:7329–7336 27274072 10.1073/pnas.1510502113 PMC4941483

[43] Rosenbloom ST, Carroll RJ, Warner JL, et al. Representing knowledge consistently across health systems. Yearb Med Inform. 2017;26:139–147 29063555 10.15265/IY-2017-018 PMC6239235

[44] Ross TR, Ng D, Brown JS, et al. The HMO Research Network Virtual Data Warehouse: a Public Data Model to Support Collaboration. EGEMS (Wash DC) 2014;2:1049 25848584 10.13063/2327-9214.1049 PMC4371424

[45] Center for Disease Control and Prevention. Behavioral Risk Factor Surveillance System. 2018 Available at www.cdc.gov/brfss/index.html Accessed 915, 2018

[46] Ogilvie D, Craig P, Griffin S, et al. A translational framework for public health research. BMC Public Health. 2009;9:116 19400941 10.1186/1471-2458-9-116 PMC2681470

[47] National Cancer Institute. Percentage computed from SEER Linkage Publications search engine using advanced search with data source-SEER-Medicare Linked Database and SEER-Medicare topic=health disparities. 2019 Available at https://healthcaredelivery.cancer.gov/cgi-bin-pubsearch/pubsearch/index.pl?source=SEERM&topic=&site=&kwOpt=and&kw=&authOpt=and&auth=&titleOpt=and&title=&yrBegin=&moBegin=&yrEnd=&moEnd=&searchOpt=and&EntryLimit=100&sortOrder=date%2Cauthor Accessed 111, 2019

[48] Institute of Medicine. Capturing Social and Behavioral Domains and Measures in Electronic Health Records: Phase 2. Washington (DC): National Academies Press (US), 2014 25590118

[49] U.S. Department of Health and Human Services (HHS). About the All of Us Research Program. 2018 Available at https://allofus.nih.gov/about/about-all-us-research-program Accessed 930, 2019

[50] Vayena E, Salathe M, Madoff LC, et al. Ethical challenges of big data in public health. PLoS Comput Biol. 2015;11:e1003904 25664461 10.1371/journal.pcbi.1003904 PMC4321985

[51] Wilkinson MD, Dumontier M, Aalbersberg IJ, et al. The FAIR Guiding Principles for scientific data management and stewardship. Sci Data. 2016;3:160018 26978244 10.1038/sdata.2016.18 PMC4792175

[52] Bartlett R, Morse A, Stanton R, et al. Consumer lending discrimination in the FinTech era. 2019 Available at https://faculty.haas.berkeley.edu/morse/research/papers/discrim.pdf Accessed 930, 2019

[53] Fuster A, Goldsmith-Pinkham P, Ramadorai T, et al. Predictably unequal? the effects of machine learning on credit markets. 2018 Available at https://cepr.org/sites/default/files/SSRN-id3072038.pdf Accessed 1010, 2019

[54] Tan S, Caruana R, Hooker G, et al. Distill-and-compare: auditing black-box models using transparent model distillation. 2018 Association for Computing Machinery, Conference on AI, Ethnics, and Society (AIES) Available at https://arxiv.org/abs/1710.06169 Accessed 1010, 2019

[55] Agniel D, Kohane IS, Weber GM Biases in electronic health record data due to processes within the healthcare system: retrospective observational study. BMJ. 2018;361:k1479 29712648 10.1136/bmj.k1479 PMC5925441

[56] Buolamwini J, Gebru T Gender shades: Intersectional accuracy disparities in commercial gender classification. Paper presented at: Conference on Fairness, Accountability and Transparency, 2018; 81:1–15

[57] Miller AP Want less-biased decisions? Use algorithms. Harv Bus Rev. July 26, 2018 Available at https://hbr.org/2018/07/want-less-biased-decisions-use-algorithms Accessed 1010, 2019

[58] Grubbs SS, Polite BN, Carney J Jr., et al. Eliminating racial disparities in colorectal cancer in the real world: it took a village. J Clin Oncol. 2013;31:1928–1930 23589553 10.1200/JCO.2012.47.8412 PMC3661932

[59] Ferryman K, Pitcan M Fairness in precision medicine. Data & Society. 2018 Available at https://datasociety.net/wp-content/uploads/2018/02/Data.Society.Fairness.In_.Precision.Medicine.Feb2018.FINAL-2.26.18.pdf Accessed 930, 2019

[60] Canner JE, McEligot AJ, Perez ME, et al. Enhancing diversity in biomedical data science. Ethn Dis. 2017;27:107–116 28439180 10.18865/ed.27.2.107 PMC5398168

[61] Morris ZS, Wooding S, Grant J The answer is 17 years, what is the question: understanding time lags in translational research. J R Soc Med. 2011;104:510–520 22179294 10.1258/jrsm.2011.110180 PMC3241518

[62] Troiano RP, Berrigan D, Dodd KW, et al. Physical activity in the United States measured by accelerometer. Med Sci Sports Exerc. 2008;40:181–188 18091006 10.1249/mss.0b013e31815a51b3

[63] Whitt-Glover MC, Taylor WC, Floyd MF, et al. Disparities in physical activity and sedentary behaviors among US children and adolescents: prevalence, correlates, and intervention implications. J Public Health Policy. 2009;30:309–334 10.1057/jphp.2008.46 19190581

[64] Belcher BR, Berrigan D, Dodd KW, et al. Physical activity in US youth: effect of race/ethnicity, age, gender, and weight status. Med Sci Sports Exerc. 2010;42:2211–2221 21084930 10.1249/MSS.0b013e3181e1fba9 PMC3242154

[65] Juarez PD, Matthews-Juarez P, Hood DB, et al. The public health exposome: a population-based, exposure science approach to health disparities research. Int J Environ Res Public Health. 2014;11:12866–12895 25514145 10.3390/ijerph111212866 PMC4276651

[66] Browning CR, Calder CA, Soller B, et al. Ecological networks and neighborhood social organization. Ajs. 2017;122:1939–1988 29379218 10.1086/691261 PMC5786432

[67] James P, Jankowska M, Marx C, et al. “Spatial energetics”: integrating data from GPS, accelerometry, and GIS to address obesity and inactivity. Am J Prev Med. 2016;51:792–800 27528538 10.1016/j.amepre.2016.06.006 PMC5067207

[68] Oyana TJ, Podila P, Wesley JM, et al. Spatiotemporal patterns of childhood asthma hospitalization and utilization in Memphis metropolitan area from 2005 to 2015. J Asthma. 2017;54:842–855 28055280 10.1080/02770903.2016.1277537 PMC6039973

[69] Baek SR, Moudon AV, Saelens BE, et al. Comparisons of physical activity and walking between Korean immigrant and white women in King County, WA. J Immigr Minor Health. 2016;18:1541–1546 26514149 10.1007/s10903-015-0290-1

[70] Den Broeder L, Devilee J, Van Oers H, et al. Citizen science for public health. Health Promot Int. 2016;33:505–514 10.1093/heapro/daw086 PMC6005099 28011657

[71] King AC, Winter SJ, Sheats JL, et al. Leveraging citizen science and information technology for population physical activity promotion. Transl J Am Coll Sports Med. 2016;1:30–44 27525309 PMC4978140

[72] Yoon S, Elhadad N, Bakken S A practical approach for content mining of Tweets. Am J Prev Med. 2013;45:122–129 23790998 10.1016/j.amepre.2013.02.025 PMC3694275

[73] Chae DH, Clouston S, Hatzenbuehler ML, et al. Association between an internet-based measure of area racism and black mortality. PLoS One. 2015;10:e0122963 25909964 10.1371/journal.pone.0122963 PMC4409363

[74] Sinnenberg L, Buttenheim AM, Patrez K, et al. Twitter as a tool for health research: A systematic review. AJPH. 2017;107:e1–e8 10.2105/AJPH.2016.303512 PMC5308155 27854532

[75] Doria-Rose VP, Greenlee RT, Buist DSM, et al. Collaborating on data, science, and infrastructure: the 20-Year journey of the cancer research network. EGEMS. 2019;7:1–11 30972356 10.5334/egems.273 PMC6450242

[76] Adams SV, Bansal A, Cohen SA, et al. Cancer treatment delays in American Indians and Alaska natives enrolled in medicare. J Health Care Poor and Underserved. 2017;28:350–361 28239006 10.1353/hpu.2017.0027

[77] Denny JC, Bastarache L, Ritchie MD, et al. Systematic comparison of phenome-wide association study of electronic medical record data and genome-wide association study data. Nature Biotechnology. 2013;31:1102 10.1038/nbt.2749 PMC3969265 24270849

[78] Dreyer MS, Nattinger AB, McGinley EL, et al. Socioeconomic status and breast cancer treatment. Breast Cancer Res Treat. 2018:167:1–8 28884392 10.1007/s10549-017-4490-3 PMC5790605

[79] Collins FS, Hudson KL, Briggs JP, Lauer MS PCORnet: turning a dream into reality. JAMIA. 2014;21:576–577 24821744 10.1136/amiajnl-2014-002864 PMC4078299

[80] Gottesman O, Kuivaniemi H, Tromp G, et al. The electronic medical records and genomics (eMERGE) network: past, present, and future. Genet Med. 2013;15:761–771 23743551 10.1038/gim.2013.72 PMC3795928

[81] Manzoni C, Kia DA, Vandrovcova J, et al. Genome, transcriptome and proteome: the rise of omics data and their integration in biomedical sciences. Brief Bioinform. 2018;19:286–302 27881428 10.1093/bib/bbw114 PMC6018996

[82] Kho AN, Pacheco JA, Peissig PL, et al. Electronic medical records for genetic research: results of the eMERGE consortium. Sci Transl Med 2011;3:79re1 10.1126/scitranslmed.3001807 PMC3690272 21508311

